# Making the Case for “Whole System” Approaches: Integrating Public Health and Housing

**DOI:** 10.3390/ijerph15112345

**Published:** 2018-10-24

**Authors:** Richard A. Sharpe, Tim Taylor, Lora E. Fleming, Karyn Morrissey, George Morris, Rachel Wigglesworth

**Affiliations:** 1 Public Health, Cornwall Council, Truro TR1 3AY, UK; Rachel.wigglesworth@cornwall.gov.uk; 2 European Centre for Environment and Human Health, College of Medicine and Health, University of Exeter, Truro TR1 3HD, UK; timothy.j.taylor@exeter.ac.uk (T.T.); l.e.fleming@exeter.ac.uk (L.E.F.); K.Morrissey@exeter.ac.uk (K.M.); G.Morris2@exeter.ac.uk (G.M.)

**Keywords:** public health, health, social care, fuel poverty, housing, air pollution, interventions, well-being, inequalities

## Abstract

Housing conditions have been an enduring focus for public health activity throughout the modern public health era. However, the nature of the housing and health challenge has changed in response to an evolution in the understanding of the diverse factors influencing public health. Today, the traditional public health emphasis on the type and quality of housing merges with other wider determinants of health. These include the neighbourhood, community, and “place” where a house is located, but also the policies which make access to a healthy house possible and affordable for everyone. Encouragingly, these approaches to policy and action on housing have the potential to contribute to the “triple win” of health and well-being, equity, and environmental sustainability. However, more effective housing policies (and in public health in general) that adopt more systemic approaches to addressing the complex interactions between health, housing, and wider environment are needed. This paper illustrates some of the key components of the housing and health challenge in developed countries, and presents a conceptual model to co-ordinate activities that can deliver the “triple win.” This is achieved by offering a perspective on how to navigate more effectively, inclusively and across sectors when identifying sustainable housing interventions.

## 1. Introduction

Evidence linking human health and well-being to poor living and housing conditions has a long history as a driver of public health policy and action. Increased awareness of the ecological and sanitation impacts on health gained momentum from as early as the mid-1800s (e.g., Friedrich Engels [1] and Rudolf Virchow [2]). Interest in health and housing increased in the early 19th century due to links with infectious diseases and changes in urbanisation (including the sanitation reform) and societal class. The Great Depression and social unrest, as well as issues such as lead poisoning, brought renewed public health attention to housing from the 1930s [3], with early evaluation studies and a number of controlled trials also dating back to the 1930s [4].

Despite this recognition, the decades immediately following World War II saw a reduction in the interest in the wider environmental and social determinants of health, and a disproportionate focus on laboratory science and curative medicine as a means to improve health [5]. The unrealistic nature of such perspectives, not least because they are potentially bankrupting to the healthcare sector, did not go unchallenged. Indeed, the socioecological model of health, so widely accepted in public health circles today, was substantially informed by Canadian Lalonde Report of 1974 [6], which challenged this approach to public health. This Canadian white paper proposed a new framework to represent the principal elements affecting health, which included one or more of four elements: lifestyle, environment, human biology, and healthcare organisation. 

Efforts to model these wider determinants of health became more detailed and sophisticated with subsequent iterations, notably the 1991 Dahlgren and Whitehead policy rainbow [7], the 1994 Evans and Stoddart health field model [8,9], and the settlement health map [10]. Barton and Grant [10] highlighted the interactions between key wider determinants of health from the natural environment, housing and housing related issues through to individual lifestyle factors. These factors are inextricably linked, with housing representing a key component of health. This complex interaction means that there is a need to engage a very wide range of policy constituencies, agencies and professions, as well as the communities and individuals affected to achieve health and well-being improvements—leading to the need for conceptual models to frame issues and be used in decision support. 

All public health activity now takes place in the context of what has been termed an ecological transition [11]. Primarily, anthropogenic damage to global systems and processes, and the attendant environmental, social and economic changes now threaten the local and global capacity to deliver health, well-being, healthcare, or equity in any of these domains in the medium to longer term. Many of the policies capable of delivering health and well-being, equity, and environmental sustainability, the so-called “triple win”, must be directed to how we live, move, and consume, especially in the urban context [12]. In particular, we need to examine policies which influence where housing is built, in what numbers, in what form, and for which communities. This is because there is an urgent need to extend beyond the current practice of delivering just the homes that are of an adequate size, quality and affordable to heat [13] if we are to deliver the triple win. These factors need to be considered in combination with the interaction and impact of wider socio-economic factors (where people are born, grow, live, work, and age), a significant issue in lower-income households. The fundamental drivers for these are the distribution of power, money and resources, which contribute to health inequalities [14]. For example, having low control in the work environment has been associated with an increased risk of coronary heart disease. There was a steep inverse association between social class (employment status) and mortality from a range of diseases in the Whitehall study in the United Kingdom. In addition to low control, there were employment grade differences in health-risk behaviours (smoking, diet and exercise), which further contributed to health and well-being outcomes [15,16]. 

While the generic topics addressed below are of public health relevance irrespective of location, this paper focuses on housing in developed countries. This is because cultural attitudes to housing, community attributes, climatic and other environmental conditions, available resources, and the administrative and legal contexts inevitably differ between the developed and the developing world. It is also important to highlight that in many developed countries, life expectancies have increased alongside higher average incomes; therefore, a greater proportion of individuals now have a choice about the quality of the home they live in. However, even in these developed countries, the health of more vulnerable populations (such lower income households) continues to be affected by sub-standard and poorly maintained housing and environmental conditions, which we discuss in this manuscript. 

**Figure 1:**
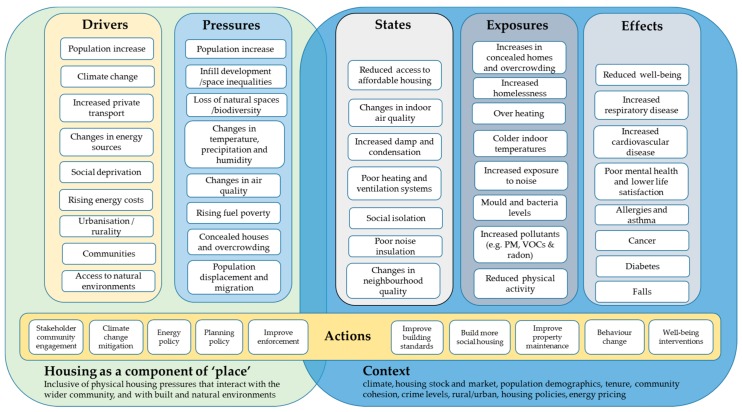
Health and housing conceptual framework using a modified DPSEEA model [126].

The following sections describe some of the drivers and pressures influencing health and well-being through housing, communities and the built and natural environment (Figure 1). Additional perspective is provided by describing key housing-related risk factors (e.g., air and noise pollution), the ability to secure healthy living environments (including housing standards through to tenure status), and housing in the context of place (e.g., communities and natural spaces). The final section draws this evidence together and discusses a conceptual model in the context of effective health and housing interventions. 

## 2. Housing, Habitation and Health

There is public and political expectation, reinforced by state involvement in housing and housing standards, that homes should possess certain basic amenities, and be designed, constructed, and equipped to provide a healthy place to live. The home should be a safe haven that is of an appropriate size, and have suitable heating and ventilation to avoid potential impacts of issues such as cold/damp homes and reduced indoor air quality. Despite the extensive evidence linking poor housing conditions with health and well-being, there is ample evidence from developed countries that poorly designed and maintained homes fail to offer protection from both the indoor hazards and the exigencies of the outdoor environment. This section focuses on some health-relevant occupant exposures affecting residents within the building envelope. Invariably these exposures are influenced by a complex interaction between the physical built environment, the type of heat and ventilation systems, and occupant behaviours and lifestyles. These exposures include indoor air pollution (physical and chemical agents), aeroallergens, cold/damp homes, overheating, and noise pollution.

### 2.1. Air Pollution

Indoor air pollution is rapidly becoming a public health priority and cause of concern [17]. This relates, in large part to the duration of exposure. In North America or Europe, people spend an average of around 89% of their time indoors [18], with approximately 69% spent in the residential indoor environment over the life course [19,20,21]. This percentage increases for homemakers and the very young, elderly, and infirm, who spend up to 90% of their time in the residential indoor environment [21]. This increases exposures to diverse biological, chemical, and physical air pollutants found in the home, which are important determinants of health regardless of whether the sources of air pollutants are indoors or outdoors.

Concentrations of indoor pollutants are influenced by outdoor air pollution (e.g., particulate matter, carbon monoxide and nitrogen dioxide) because outdoor air pollution can be effectively transferred into the indoor environment where concentrations may be between two and three magnitudes higher [22]. In fact, the combination of outdoor and indoor pollutants inside the home means that concentrations can be as much as ten times higher indoors than outdoors because of the additional presence of internal sources [17,23]. 

Indoor pollutant sources include: the by-products of combustible processes (e.g., particulate matter, carbon monoxide, sulphur dioxide and nitrogen dioxide from cooking, heating and environmental tobacco smoke), radon, lead (e.g., water pipes and lead paint), chemicals including volatile organic compounds (e.g., aromatic hydrocarbons, aldehydes, aliphatic halogenated hydrocarbons, and terpenes resulting from building products and household furnishing/products), and formaldehyde (e.g., from insulation off-gassing). The impact of these air pollutants on health depends on the level of exposure, which is in turn influenced by the type of heating and ventilation systems, as well as the type of materials and products used during construction, and other household products introduced into the home by residents [24]. In terms of combustible by-products, it is generally thought that air pollutant concentrations are highest in homes with resident smokers [25]. If properly designed, installed, and maintained (usually a function of building regulations), the pollutants resulting from cooking and heating generally fall within acceptable levels [24]. The off-gassing from indoor materials are an important exposure; and can be released from wood-based products, paints, floor finishes, glues, consumer products and freshly dry-cleaned clothing are also each implicated [26]. However, unless a home suffers from dampness and/or new materials are introduced into the home (e.g., chemicals, paints and furnishings), these emissions generally decay over time [24]. This explains why concentrations of formaldehyde and volatile organic compounds have been found in higher concentrations in newer homes [27]. The emissions resulting from materials (e.g., floor-levelling compounds, plaster, and furnishings including carpets) are a particular risk factor for asthma [28]. Also of interest are naturally-derived pollutants and those from antiquated building materials. For example, increased exposure to some agents such as increased radon and asbestos can have synergistic effects on the resultant health outcomes when combined with smoking [29,30].

Increased exposure to these physical and chemical agents in the indoor environment increases the risk of: respiratory tract infections, chronic obstructive lung disease, asthma, allergic diseases, lung cancer, cardiovascular events, and cognitive decline. The resultant impacts on health depends on a complex interaction between building characteristics (e.g., build type and age), and resident behaviours (e.g., smoking, use of fungicides/biocides, cooking, heating and ventilation patterns) [31,32]. Consequently, reducing concentrations of these indoor pollutants represents an important risk prevention/reduction measure [33] for a range of health effects. Moreover, cold and damp conditions in homes further modify the indoor concentrations of these pollutants, as well as indoor aeroallergens.

### 2.2. Aeroallergens

Exposure to indoor aeroallergens is influenced by the presence of pets (e.g., cats and dogs), and pests (e.g., cockroaches, mice, and rats) [34]. The presence of indoor dampness (resulting from water leaks, rising damp and condensation), which affects 16% of European homes [35], leads to increased volatile organic compounds (degradation of building materials) [36], bacteria, and the proliferation of house dust mites and mould growth (spores, hyphal fragments, mycotoxins and cell-wall b-glucans). The extent of these pollutants depends on a range of factors. These include relative humidity which when elevated can give rise to surface condensation, rising/penetrating damp and water introduced by plumbing leaks or flood events. Ambient/surface temperatures, ventilation and sunlight also influence hygrothermal conditions and by extension the proliferation of allergens in the home [24]. Increased relative humidity is an important factor influencing the concentrations of bacteria, house dust mite allergens, chemical interactions and ozone, which also influence health and well-being outcomes [37]. Predictably, these important sources of indoor aeroallergens are each risk factors for the development and exacerbation of allergic diseases (e.g., eczema, allergy and asthma) [26,34]. 

Aeroallergen levels are influenced by the type and location of a property, structural building maintenance levels, heating and ventilation, and resident behaviours (e.g., drying washing indoors and presence of pets) [24]. Importantly, the health outcomes experienced by occupants depend on the timing and concentration/diversity of indoor aeroallergens [38]. For example, early exposure to some aeroallergens can result in divergent health with possibly a protective effect (the hygiene hypothesis) in some populations [39,40,41,42,43]. However, emerging evidence implies a need to standardise protocols for characterising indoor air quality, the use of consistent measurement strategies, and the development of tools for policy implementation [44].

### 2.3. Cold and Damp Homes

Another important factor influencing the presence of dampness and the resultant health effects in vulnerable households is occupancy of a cold home. Cold homes may be a result of structural issues (particularly in old houses) or “fuel poverty”. Fuel poverty has become an emergent topic over the last decade and applies to households that are unable to adequately heat and ventilate their homes (due to the cost of fuel and/or energy efficiency of the home). Fuel poverty is a distinct societal public health concern, affecting between 6% and 34% of households [45], although patterns in fuel poverty vary both spatially and socially between and within countries [46,47,48]. Fuel poverty is influenced by the complex interaction between income, housing conditions, situational and contextual factors, attitudes, values, and barriers. In combination these factors influence people’s ability to heat the home, access help, or change heating behaviours [49]. The impacts of fuel poverty on the indoor environment depend on resident behaviours, which may themselves be a function of risk perception and knowledge (e.g., in relation to use of the heating system), levels of occupancy, the efficiency and controllability of the heating system, and building characteristics (e.g., building age, type and tenure) including the location and orientation of a building [24].

Fuel poverty is a public health priority because it poses a significant risk to occupant physical and mental health and well-being, including increased cold-related morbidity and mortality [45,50,51,52,53]. The impacts of cold and damp homes illustrate the complexity and importance of the interactions between multiple social, cultural and economic factors influencing health and well-being outcomes.

### 2.4. Overheating of Homes

In contrast to the impact of cold and damp homes, properties can overheat, which in turn can have an impact on resident health and well-being. As with cold indoor conditions, overheating depends on many interrelated factors such as occupant susceptibility to heat, their behaviours (e.g., use of ventilation and time spent indoors), and the building location and its characteristics (such as the extent of insulation and building materials) [54]. Measures which increase household energy efficiency levels without considering the potential consequences on health (e.g., overheating and reduced air quality) [55] and climate change [54] pose a continual risk to residents health and well-being. Conversely, a lack of roof insulation, as well as a lack of mobility, susceptibility to heat (e.g., a pre-existing illness), location, and temperatures around the property all contributed to the death of older adults during the 2003 heat wave in France [56]. Improved housing and care of the elderly at home can help reduce the risk of overheating [57]. 

### 2.5. Noise Pollution 

Noise exposure is generally considered separately to that of air pollution [58], although there can be a synergistic effect in urban areas [59]. Environmental noise is one of the most prevalent environmental hazards and an independent risk factor [60] for cardiovascular morbidity and mortality [58,61,62]. Noise pollution is estimated to affect one-third of the global population [63]. Noise in the home generally comes from external environmental sources with nocturnal exposure being a public health concern because of its impact on sleep even at relatively low levels [64,65]. Much external noise can come from road and rail traffic, air transportation, and occupational and industrial activities, as well as individual or community-noise-level exposures (e.g., amplified music, recreational activities, and firearms) [59]. In many countries, excessive noise exposures come from predominantly occupational settings (i.e., noise emitted from commercial/industrial premises) and transport, with road traffic being one of the top three stressors affecting public health [59,66]. It is also thought that excessive ambient noise exposures can also lead to an increased use of headphones where loud music can in turn impair hearing (and hearing-related symptoms are associated with diminished health and well-being) [67].

This means these individuals are at increased risk of noise-induced hearing loss, heart disease [59], and diabetes [66], as well as psychophysiological effects (e.g., annoyance, reduced performance, and cognition skills), and changes in social behaviour [58]. Of concern is the impact of night-time noise, which is likely to be associated with increased risk of cardiovascular disease and stroke in the elderly for example. The impact on sleep is thought to have a greater impact on health and well-being than daytime noise exposure [65]. The effects are thought to result from disturbing sleep patterns, where reduced noise levels and reverberation lead to an increase in the amount of deep sleep and to reduction of nocturnal arousal events [64]. In many cases, the causal pathways are unclear, requiring further research involving the quantification of the impacts of merging noise sources (e.g., high speed rail, wind turbines) [65]. 

In lower income households, the extent of domestic exposure to all of the health-relevant aspects of the home environment discussed above is a function of a household’s ability to secure healthy housing in a location where they wish to live. Typically, people do not choose unhealthy housing for themselves and their families when healthy alternatives are available. There is an established interaction between income, resultant exposure to outdoor air pollutants, and associated morbidity and mortality in many cities [68]. Income and air pollution are key determinants of health. Nevertheless, while those with lower incomes have been found to be at higher risk in urban areas such as Ontario in Canada [68], it is also possible that higher-income households and young professionals choose to live in more urban areas where air pollution is at its greatest. 

For the individual household, barriers to access are formed by the interaction of many factors. These include choice, affordability (where housing construction and intervention standards and market-related issues are likely to be important), tenure (where an adequate tenure mix and the barriers to access for different tenure categories are critical), and the size and accommodation profile of housing stock (which among other effects can influence levels of overcrowding in a community). Some of these factors are explored in greater detail below.

## 3. Factors Which Can Influence the Individual’s Ability to Secure Healthy Housing

As described above, the availability and access to healthy housing is driven by a number of factors. Policy measures, including housing standards, may address some of these issues—although the impact on housing prices may further compound health equity among lower-income households. The housing market is marked by market failure, with examples including homelessness in a time when houses stand empty, and the environmental impacts of new house construction and geographical immobility leading to housing shortages in certain locations. In this section, we investigate different factors that may affect the availability and accessibility of healthy housing, with a focus on housing standards, affordability, tenure, and overcrowding. 

### 3.1. Housing Standards

Existing legislation and statutory guidelines aim to build and maintain healthy homes that overcome potential health risks. Most countries have a range of building regulations and statutory guidance covering the design of buildings, materials used, ventilation and heating systems, water and energy conservation, fire safety, sanitation access, and the prevention of falls [69]. At the same time, housing supply policy and construction are increasingly being affected by a global shift towards more sustainable building design standards, which encourage greener development, including the use of sustainable sites, water and energy efficiency, material use, indoor environmental quality, emissions, and maintenance [70]. In response to the various oil/energy crises and climate change risks, sustainable building practices to create more resource-efficient models of construction, renovation, operation, maintenance, and demolition gained momentum in the late 20th century across the United States and Europe. 

The legislative framework differs with country-specific examples including Part 1 of the Housing Act 2004 in the United Kingdom, [71] and standards such as the Housing Health and Safety Rating System [72]. There is also the Healthy Housing Standard in the United States, which identifies hazardous living conditions and provides recommendations for household systems from heating and ventilation through to the control of mould, pests and chemicals such as radon, lead, formaldehyde and asbestos [73]. Standards vary because of different housing stock, and different populations and health issues associated with the built environment. These may include, for example, the installation and maintenance of air conditioning and/or mechanical ventilation systems in the United States [74], which are currently not as widely installed in the United Kingdom. However, building codes and national regulations governing new developments are often vague, and do not consider the characteristics needed to provide adequate shelter from the perspective of human health and well-being [69]. In some cases, the legislative framework fails to capture home improvements and changes to the building fabric such as those to improve the energy efficiency of homes, and their unintended and intended impacts on the housing stock and on health and well-being. Consequently, these are performed without scrutiny from planning and building control, for example.

While improved housing policy can help raise housing standards, legislative approaches fail to raise the standards of living conditions particularly in lower income households with significant social deprivation. Also, state involvement to create and enforce housing standards almost inevitably raises the cost of providing housing, which can affect the affordability of housing for lower income households. Despite the legislative framework and the adoption of highly developed technologies, materials and construction styles, poor housing remains a major cause of ill-health worldwide including in developed nations. In New Zealand, the Housing, Health and Health Study highlighted a range of housing-related risk factors, which are common to other countries such as the United Kingdom and United States. These include health risks associated with housing such as deprivation, overcrowding, tenure, and affordability [75]. Being able to afford your home versus living in rented accommodation (e.g., public housing) or within the private rental sector can influence individual and community health and well-being [76]. This means that improved fiscal mechanisms are required to overcome these underlying socio-economic factors impact the home environment.

### 3.2. Housing Affordability

Being able to afford your home is a key factor influencing health and well-being [77]. Outside of the public health arena, debates about planning regulation and housing outcomes have achieved considerable political resonance in developed countries over the past decade [78]. In countries where housing supply has failed to keep pace with demand, home purchase has become increasingly unaffordable for low- and moderate-income earners [78,79]. Rising energy prices, food price inflation, and increased rents further compound the impact of higher house prices, as people struggle to raise the deposit to buy their own home in countries such as the United Kingdom. 

Housing supply, for example is relatively flexible in North America and some Nordic countries when compared to European markets [80]. In Australia, ongoing changes to the planning system itself have created uncertainty, and distract from the range of positive policy levers that might be used to promote housing supply and affordable homes (although this may differ from European housing supply models and regulations) [78,81]. Changes in policy can also have an impact on housing supply. For example, an increasing shift towards infill development (building within existing property boundaries), coupled with a decreasing provision of government-owned social housing in Australia is placing severe pressure on housing affordability [82]. Affordability of homes is modified by housing tenure, including home ownership and the private or public rental sectors, which are largely driven by socio-economic status and ability to secure an affordable home. Other potential measures such as rent control to improve accessibility may have unintended consequences, reducing the available supply and worsening housing conditions [83]. 

### 3.3. Health and Housing: Housing Tenure 

Higher levels of psychological distress are found in those renting their home; while those who own their home without mortgages have the lowest distress levels [84]. Home ownership is often assumed to have universal health benefits compared with those residing in the rental sector, including lower risks of ill-health, general health, anxiety, and depression [76]. Ownership enables higher income households to choose the type of property they wish to live in, which may be a choice to buy or to rent a property. Owning your home helps households move up the housing ladder, work harder, and save more money, which can improve the accumulation of home equity (the amount of money tied up in the home) and provide a range of other individual and societal benefits. These include the accumulation of equity, and an improved ability to maintain spending in the wake of an adverse income or expenditure shock (e.g., the home can be used as a collateral asset against which to borrow). Societal benefits include stable community environments and a reduction in crime [85].

However, health and well-being outcomes among home owners can be significantly affected by home equity, affordability, and unemployment “shocks”. While reducing mortgage rates can help improve affordability, increasing mortgage interest rates can have an adverse impact on financing and the affordability of a household [86]. Sharp price increases in both owning and renting a home (e.g., resulting from state intervention and improved standards and/or market pressures) mean that a higher proportion of a household total income is needed. This reduces the ability for saving/getting on the housing ladder, and can lead to financial problems and mental health problems [87]. It is also important to highlight that the size of this housing sector can mask enduring problems such as short spells of poverty associated with socio-demographic and spatial differences [88]. 

Even in countries where residents residing in rented accommodation have better protection such as rental rights (e.g., in Germany), renting a home has been associated with poorer self-rated health. This is mediated by the need for household renovation, the perception of air and noise pollution in the local area, and more distant relationships with one’s neighbours [89]. An increasing public health concern is the welfare of families residing in multiple occupation houses (i.e., families share public spaces such as the bathroom and kitchen) in the United Kingdom [90]. However, other models of public and multi-family housing such as those in the United States have been found to result in improved physical and psychological health [91], which may be due to different legislative control and behaviours. 

Other important types of accommodation that can influence health and well-being include: temporary dwellings such as park homes or caravans, which are mobile and moveable (although some park homes may have a reasonably long life, and are not readily moveable) [92,93]; and adapted or specialist housing such as supported or sheltered housing and temporary accommodation (e.g., temporary in terms of using a hostel or bed and breakfast), often used for those who are homeless (i.e., those with no permanent accommodation) across all European countries [94]. These precarious housing circumstances and associated health impacts [95,96,97] can reduce life expectancies among these vulnerable populations, particularly among young adults living at home and the homeless [98,99]. The homeless are a particular concern because of the increased risk of both poorer physical and mental health outcomes experienced by this vulnerable population [98]. Resultant health outcomes influenced by tenure status can be further modified by a range of issues such the location of the home or “place” and the distance to employment [100], which in turn can have an impact on stress levels relating to commuting. Other important factors include risk factors such as overcrowding within rental or temporary accommodation.

### 3.4. Over-Crowding (A Function of Affordability and Tenure)

Household tenure, household overcrowding, and housing affordability are highly intertwined, but are among the most commonly reported pathways for poorer physical and mental health outcomes [101,102]. The impact of socio-economic deprivation and unaffordable housing discussed above can lead to poor housing conditions, overcrowding, increased numbers of concealed households (i.e., families living together in the same dwelling), and homelessness [103]. Overcrowding (also referred to as “crowding”) refers to the number of people sleeping in a dwelling that contravenes the room or space standard for a given dwelling [104]. Overcrowding is further exacerbated by: the decline in the overall size of homes, reduction in social (or public) housing, the rise of single-person households, changes in occupation thresholds set by private and social or public housing landlords, and the trend towards building smaller properties (internal and external spaces) [105]. 

Poor housing conditions combined with overcrowding and associated indoor air quality issues (e.g., increased dampness [24]), have been found to have an indirect (e.g., increased stress) and direct relationship with physical and mental health problems [106]. This can be exacerbated by lower educational attainment in later life [107] and the type of property. However, the specific mixture of risk factors varies from country to country, depending on factors such planning legislation, and the design, variable heating, and ventilation rates of properties [108]. The interaction between affordability, tenure and issues such as overcrowding can have a significant impact on households living in fuel poverty. While these housing issues persist and are important for health and well-being outcomes, they are also an essential component of “place”.

## 4. Housing in the Context of “Place”

In the previous sections, we identified specific aspects of the domestic indoor housing environment which continue to represent a threat to health and well-being for many in the developed world. Consequently, the lack of affordable healthy housing and poor housing conditions remains a significant public health burden. Moreover, social patterning in housing, which consistently results in society’s most disadvantaged persons and communities enduring the poorest housing conditions, makes housing a major contributor to health inequity. In Section 3, we explored a wider set of issues affecting an individual or family opportunities to access a healthy home in a suitable location and within their means. Here, we consider the implications for housing policy in the context of place, and the importance of the surrounding environment and community structures. 

Among the greatest impacts of the socioecological model of health was the acceptance of a more complex context for public health policy. Based on these socioecological models, individuals’ health and well-being are products of a complex interaction between economic, social, cultural influences and individual characteristics (including behaviour). These factors must be reflected in how health and housing challenges are framed, analysed and addressed. With specific reference to housing, this requires consideration of the attributes of the homes themselves and the factors governing access to healthy homes, but also the total lived experience of a household in the context in which it is located. Drawing attention to the health risks associated with the social structure and ecology of place or surrounding neighbourhoods can help to promote the development of community level interventions [109] in the context of place.

The term “place” has been adopted to capture the integration of the social, economic, environmental, cultural, and historical aspects of a location. It is argued that “good places” are more likely to produce healthy, happy, productive people and communities (who, in turn, collectively reproduce good places). Thus, housing, in its widest sense, can be seen as a component part of the site and of the system by which health outcomes, good and bad, are generated and are often reproduced over the life course and from generation to generation [110]. In practical terms this means that health and housing are a function of location, neighbourhoods or communities, the availability of amenities, transport etc., as well as the natural environment and environmental factors such as access to green/blue space, and noise and pollutant levels. The following section provides an overview of the importance of the healthy neighbourhoods and outdoor spaces.

### Neighbourhoods, Communities and Access to Natural Spaces

The natural and built environments are important risk factors influencing public health outcomes. For example, neighbourhood attributes such as residential density, safety from traffic, recreation facilities, street connectivity, walkable environments, and the location and type of stores are all important factors influencing health and well-being outcomes [111]. Consequently, since the early 2000s there has been a drive towards access to more natural environments and towards urban environmental sustainability as a potential approach to addressing global climate change and rapid urbanisation. This is increasingly being mainstreamed in policy-making [112]. 

Sustainable communities involving well-designed buildings and outdoor spaces can enhance the long-term health and well-being of those who use them regularly, reduce the risk of falls, promote physical activity, and reduce social isolation [113,114]. Improving the wider neighbourhood and environment (for example through increased access to parks and open spaces) has been estimated to reduce healthcare treatment costs by £2 billion in the United Kingdom [115], while helping to reduce crime and residents’ perceived fear of crime [116]. 

To achieve these benefits, requires a shift towards new alternative forms of sustainable housing development where developers, investors, landowners, and the communities work together to achieve more liveable neighbourhoods [117]. This should encompass the provision of improved quality of the natural environments and access to “green space” and/or “blue space”, which help to promote human health and well-being [118,119,120,121,122]. Local governments play a vital role in protecting, maintaining and improving local green spaces, and can create new areas of green space to improve access for all communities [123]. To achieve these improvements within and outside the home requires greater collaboration between multiple sectors influencing health, housing, communities, and natural environments.

## 5. Discussion

Current evidence concerning the home environment, the place in which it is situated, the community, and wider ecological changes in terms of both the built and natural environments make a clear case for more holistic and integrated approaches to improving health and well-being through housing. This is not solely about placing people in more “healthy homes”. Future strategies must take account of wider social, economic, environmental, and policy factors. This includes policies which influence issues of affordability and equity, tenure, access, housing, and sustainability standards, and how these interact with resident behaviours, lifestyles, and local communities as well as built and natural environment. 

To achieve more salutogenic outcomes, these must be addressed alongside improvements in the quality and access to healthy built (e.g., indoor environment) and natural environments, as well as addressing issues of air pollution, noise, and level of environmental incivilities. These are all health-relevant, especially in an era where we understand the socioecological dimensions to the relationship between humans and their environment, which influence responses such as stress biology [124]. From a public health perspective, these are all important socioecological determinants of health where housing should be seen as a component of “place”, which considers the interactions between the multiple dimensions of modern society, including economic, social, cultural, and historic influences. The complex interaction between these factors influences the health and well-being outcomes of individuals and their communities across the life course. 

Importantly, these emerging risk factors will have important implications for countries experiencing comparable issues with substandard housing and environment conditions [3,74,125]. There are clear opportunities for public health intervention (discussed below), however, these must not be considered in isolation from these wider determinants of health [10] and potential unintended consequences. This requires the involvement of multiple agencies and sectors because of the overlapping nature of individual lifestyle/community factors, and the built and natural environments. 

Investigating these pathways leading to health effects via a modified DPSEEA model (Drivers Pressures Exposures Effects Actions) [126] provides an opportunity to identify key factors to inform future collaborations and help develop more sustainable health and housing (Figure 1). This conceptual model enables the identification of higher-level drivers that lead to pressures, with resulting impacts on the state of the environment that influences exposures and ultimately leads to health effects. Delivering more multifaceted interventions (i.e., addressing both existing and new builds) as well as the essential engagement of individuals and communities and across sectors (i.e., housing, education, transport, health, environment) are all necessary to develop guidelines that extend beyond the current best practice of delivering dwellings that are of an adequate size and affordable to heat [77]. However, there is conflicting evidence on which interventions work to lower the societal and economic burden of poor living environments. The following provides an overview and discussion around prior housing interventions and their effectiveness in improving health outcomes.

### 5.1. Home Improvements

Well-designed energy efficiency or home improvement/adaptation interventions (i.e., combination of draft proofing, insulation, glazing and heating upgrades) can consolidate the meaning of the home as a haven, strengthen the householder’s perceived autonomy, enhance social status, and improve home satisfaction and financial considerations, quality of life, and thermal comfort [127,128]. These interventions can improve indoor air quality [129], and reduce risk of mould growth and the proliferation of house dust mites [130,131], as well as help alleviate fuel poverty and increase sustainability [132].

Consequently, these improvements can prevent falls and other injuries, and result in improvements in general health and well-being, with respiratory, psychosocial (e.g., positive perceptions of internal improvements and home security), and mental health benefits [77,133,134,135,136,137]. This may be particularly true in those cases where the improvements are targeted at those with the greatest needs, such as individuals with chronic respiratory disease [77], especially when combined with the promotion of mixed exercise, education, and assistive technology in the home [138]. Despite the limitations of the studies (including their short follow-up), energy efficiency and/or improved housing quality standards may reduce the risk of being admitted into hospital for cardiovascular and respiratory problems [139,140].

To be effective, these should account for potential unintended consequences of some housing improvements [141]. This means that not all interventions have resulted in long-term improvements, with for example some residents feeling that the process of receiving the intervention was stressful [128]. Furthermore, some interventions have resulted in only short-term benefits [142], or actually reduced indoor air quality and increased health problems [13]. There is also recent suggestive evidence that historic housing improvement programmes (e.g., for those that are poorly designed and those that do not deliver “whole house” solutions) may increase the risk of hospital admissions for cardiovascular and respiratory problems [143]. These potential unintended consequences of home improvement programmes [141] could be avoided through improved housing quality standards [140] that adopt a more holistic approach, considering individual lifestyles and interactions with communities and both the physical built and natural environments. 

Within the existing housing stock, technical solutions (such as passive ventilation with heat recovery or mechanical ventilation with heat recovery) may help improve indoor air quality. However, there needs to be a trade-off between primary energy, carbon dioxide emissions, household consumer energy price, and exergy policy. It is thought that a low specific fan power will make heat recovery advantageous within the home environment [144]. Moreover, to make housing interventions more effective, it would be beneficial to deliver technical solutions alongside resident training and professional housing inspections to identify the most sustainable solution within individual households. This means that future interventions need to consider: (1) potential unintended consequences; (2) the impact of an intervention on the household (e.g., levels of stress); (3) the longer-term benefits of “whole house” solutions; and (4) that interventions may only be effective when combined with resident training.

This is important to consider because the adoption of “whole house” (e.g., the incorporation of resident training and physical improvements to the property with improved heating and ventilation) systems such as the “green public housing” or “healthy homes” initiatives [145,146] or those in New Zealand [147,148,149] consider resident behaviours and the dwelling, and have led to demonstrated improved health and well-being outcomes [146]. Well-designed salutogenic household improvements household energy efficiency and greater ventilation rates (with heat recovery and heating systems) such as those in the US ASHRAE (American Society of Heating, Refrigerating and Air-Conditioning Engineers) standard [150], and in particular, resident training, community engagement [128], and improved awareness among housing and health practitioners [151], have the potential to deliver a range of co-health benefits [152]. To achieve more sustainable outcomes, home retrofit improvements need to be addressed in conjunction with improvements to the wider neighbourhood and local natural environments, which intersect with communities and place (i.e., a “whole systems” approach).

In terms of economic analysis of home improvements on health, little has been done. A systematic review of this topic showed only four studies to have undertaken an economic analysis [153]. Three studies applied a cost–benefit framework, and only one study a cost-effectiveness approach. The three cost–benefit studies did not include monetary valuation of all major outcomes and the cost-effectiveness study did not present the results fully. Those results suggested that improved housing was not less costly, nor was it more effective. Given the multiple benefits of improved housing, there is a clear need for further research into the economic analysis of interventions using a cost–benefit framework. 

### 5.2. Long-Term Prevention, and Community Involvement

To achieve “whole systems” approaches, more multifaceted housing [154] and wider community and individual-level interventions [155] are needed. These need to incorporate combined plans to support the homeless [138,156], urban regeneration [138,157] with mixed housing and land use [158], “age friendly” environments [159,160,161], and open spaces with community access [158]. These need to be designed in a way that avoids potential issues such as the trend in delivering smaller living environments and urban infill, which contribute to issues such as space inequalities. Wider community development strategies [162] and improving access to more natural environments across the social gradient have the potential to improve health. Neverthless, while wider community interventions to build community resilience and reduce inequalities [163] have improved chronic health problems [164], these interventions to date have failed to address individual healthy behaviours such as diet and physical activity [165]. 

Delivering increased access to natural environments can increase community cohesion (e.g., reduce social isolation), mental and physical health, and the general well-being of residents [118,119,120,121,122]. However, the specific mechanisms by which exposure to and interaction with green/blue space affect health and well-being are still not well understood [118], and could result in a number of unintended consequences (e.g., fear of crime or poor perception of the environment) if not properly co-designed [118,166,167]. While the evidence is mixed, this raises again the essential need for community participation to create more socially cohesive environments [168]. Importantly, delivering home improvements alongside neighbourhood regeneration with community engagement (with increased access to nature spaces and age friendly environments) has been found to improve community resilience and health outcomes [4,136,169]. 

Furthermore, improving areas such as education, occupational skills and economic status can help alleviate individual and community deprivation risk factors, and improve social functioning and physical and mental health outcomes over the life course [170]. This also requires alternative funding models, and fiscal incentives for homeowners/land lords because they are less likely to invest in home improvements under the current home improvement programmes [171]. To achieve these public health outcomes, future multifaceted housing and wider community and individual interventions require more careful evaluation. More complex modelling is needed to understand how complex direct and indirect risk factors (Figure 1) influence all dimensions of health and well-being [31,172]), particularly with respect to housing and place that are resilient against the risks posed by changing climate [172,173,174]. 

### 5.3. Implications

Resolving these complex societal, economic and environmental issues requires multi-faceted approaches including sustainable building design, and particularly community engagement and participation and social inclusion throughout the intervention processes and beyond to achieve sustainable and positive change [166]. Some evidence suggests that these should include a shift to more person- and community-centred ways of working in public health and healthcare, covering key themes such as strengthening communities, opportunities for volunteering and peer roles, fostering collaboration and partnerships, and access to community resources [175]. These should be delivered alongside a change in current and future housing and wider environmental policies, including housing affordability, suitability, appropriateness, equity, environmental sustainability, and security [176]. 

The “whole systems” approach requires greater shift in policy and the integration of stakeholder resources and responsibilities to achieve positive outcomes. In the United Kingdom for example, the potential shift towards integrated care systems (i.e., new collective responsibility for resources between health sector and local authorities) may provide the mechanisms to achieve this more holistic and joined up approach to improve community and individual health and well-being through housing [177]. 

However, more integrated built and natural environment policies are needed to facilitate a shift towards more holistic “whole systems” approaches. While improvements in spatial planning provide a framework for designing healthier built and natural environments [178], this fails to account for the existing housing stock and wider communities. This is important because there is a clear need for more sustainable urban regeneration, which must account for both urban and rural areas [108]. A shift towards integrating health and well-being through planning [178], transformation partnerships [179], and the development of cross-sector Memorandum of Understandings [180], while incorporating both communities and decision makers (including those at the built and natural environment level) at the policy level [181] has the potential to achieve these “whole systems” approaches.

## 6. Conclusions

Improving population-level health and well-being through housing should not be addressed in isolation from wider individual, community, and environmental factors influencing health and well-being. While some interventions can achieve positive public health outcomes, these sometimes fail to account for other potential unintended consequences that occur when interventions are delivered in isolation of wider community and environmental considerations. 

To overcome this, future interventions must consider housing as a component of “place”, while considering social, economic, physical, cultural, environmental, and historical differences among communities. This requires more integrated holistic approaches to spatial planning and urban and natural environment regeneration, with improvements within the home (including behavioural factors), the community, and natural spaces. These have the potential to deliver a range of public health outcomes when combined with community engagement. This “whole system” approach that targets these wider determinants of health and includes continuous community and policy maker involvement throughout the process has the potential to improve health and well-being outcomes at the population level. To be effective, this requires a greater shift towards integrating health and well-being with housing and the larger context of public health, requiring changes within both built and natural environment policy and practice.
